# Diseases of the Digestive Organs, as the Primary Causes of Consumption and of Many Other Diseases

**Published:** 1893-03

**Authors:** W. L. Morgan

**Affiliations:** Baltimore, Md.


### INTERNATIONAL HAHNEMANNIAN ASSOCIATION MEETING OF 1892.

---

### DISEASES OF THE DIGESTIVE ORGANS, AS THE PRIMARY CAUSES OF CONSUMPTION AND OF MANY OTHER DISEASES.

W. L. Morgan, M. D., Baltimore, Md.

The digestive organs constitute one of the most important groups in the human organism. These are the salivary glands, œsophagus, stomach, duodenum, jejunum, ileum, appendix, the ascending, transverse, and descending colon, with the hepatic, splenic, and sigmoid flexures; and the rectum, liver, spleen, pancreas, mesenteric glands, and thoracic duct. These are the primary organs, whose office is to prepare material to build, form, and sustain every organ, tissue, and function, even the vital force and the brain, which is the organ of the mind. The digestive organs are oftenest over-worked, strained, and are seldom allowed to rest; they are the victims of abuse from every intemperate habit or act, including the indulgences of the table and of fashionable life. When we see the amount of work these organs are required to perform and their delicate structure, we cease to wonder at the great number and severity of diseases to which they are subject.

With few exceptions the diseases of the stomach will apply equally to the entire digestive tract from aphthous sore mouth to piles, fissures, prolapsus recti. The diseases of the stomach are more numerous than is supposed at first thought. It is generally and correctly understood that all irritation, all kinds of pimples, eczema, pustules, infectious rashes, such as measles, scarlet fever, itch or scabies, and suppurating ulcers, when suppressed from the skin by salves, ointments, plasters or medicated washes (and by many supposed to be cured), generally transfer their openings to the mucous membrane of some internal organ, usually the digestive tract, and especially the stomach.

Think of a corroding ulcer on the leg, how it burns, pains, itches, and the corroding pus that causes an eating sore wherever it touches the skin, in which the modern scientists find dangerous bacteria with long horns, sharp claws, ravenous appetite, and a capacity for reproduction by millions per day!

But we will leave them on the shelf and reflect that many of these conditions may be in the stomach, and, in addition to them, catarrhal sores and thickening of the membranes, which cause unhealthy secretions. Superficial and perforating ulcers, cancers, all produce poisonous secretions, which are at once mixed with the ingesta, along with other degenerative substances mentioned below, and with the digestive fluids of the stomach; and these are fused together with imperfect digestive fluids from a diseased organ, which may be too acid or too alkaline, or deficient in vital properties, which the chemist cannot determine. Besides these lesions and the excruciating pains suffered by the patient, there are cramps, strictures, spasms, atony, flatulence, and many other morbid conditions caused by perversion of the vital force, intussusception of the bowels, incarcerated flatus, parasites, nausea, vomiting, hemorrhage, etc. The descriptions of the pains and nervous states attending the above diseased conditions are not necessary to the object of this paper and are omitted.

For convenience, I will call attention to some of the most important diseases of the digestive organs in the order given in Volume I, of Arndt's *System of Medicine*, namely: œsophagitis, dysphagia, stricture of the œsophagus, dilatation of the œsophagus (œsophagocele), perforation and rupture, morbid growths, neurosis, spasmodic stricture and paralysis of the œsophagus.

Then following the canal we find the stomach the seat of many more of the most painful and distressing affections that are known, viz: atonic dyspepsia, cardialgia, gastralgia, gastrodynia, cramps, acute gastric catarrh, gastritis toxica (poison), chronic gastric catarrh, ulcers, superficial and perforating cancer, vomiting of blood, stricture of cardiac orifice, obstruction of pyloric orifice, dilatation, stenosis, hypertrophy, tumors, abscesses and ruptures of the wall of the stomach, albuminoid, fatty and tubercular degeneration. Add to these the numerous functional derangements, such as excess or deficiency in the secretion of the digestive fluids and any of the vitalizing agents. In this way we will pass along the entire length of the intestinal tube, and the same imperfect secretion of fluids necessary in the proper preparation of the food to nourish the system must make imperfect digestion and imperfect chyle, which produce diseases of the mesenteric glands and lacteals, and make imperfect blood. This must, of necessity, cause imperfect tissue, defective organs, susceptibility to infectious and other diseases, inability to throw off effete matter. Hence, the result is tuberculosis, overworked and diseased liver, impure bile. Thus starts another cycle of imperfect digestion for the formation of more bad blood, numerous kinds of headaches, eyeaches, toothaches, neuralgia, boils, carbuncles, abscesses, felons, neurosis, vomiting, periodic bilious attacks. Then diarrhœa may set in, with kidney and vesical irritation, hemorrhoids, fistula (and if a female with prolapsus, versions, flexions, leucorrhœa) the patient may become hysterical, nervous, fretful, contrary, melancholy. The stomach may be swollen, sore and distended with flatus, the pancreas may be enlarged, and both, by mechanical action, interfere with the great solar plexus of the sympathetic system of nerves, and give rise to hypochondriasis, cause the children to be scolded and whipped, bring about the family quarrel, separation, and lawsuit. All these, and even suicidal mania, homicide, suicide, and a thousand other troubles might be averted by securing a thoroughly healthy condition of the digestive organs.

I will now follow the process through which the nutrient material passes, together with its relation to the healthy or diseased mucous membrane and other organs of digestion. When liquid is taken into the mouth, it is passed at once into the stomach, with the saliva which it has excited; but with solid substances it is quite different, as in the after processes it is necessary that it be reduced to a semi-liquid state. For this purpose the irritation from the presence of the solid substance in the mouth calls on the salivary glands for saliva to incorporate with the food as the preparation for digestion, after it is retained in the mouth a reasonable time undergoing the process of mastication till sufficiently mixed with the fluid from the salivary glands. It is then ready to be transferred to the stomach for the first stage of digestion. If the mouth, salivary glands, pharynx, or œsophagus, one or all, are in a diseased condition, this disease may be infused into the ingesta. If the salivary glands are in an inflamed or diseased condition, it is fair to suppose that the fluid will be either impure or deficient in quantity, and that the ingesta will pass to the stomach badly prepared—that is, lacking in properties or charged with impurities or deleterious substances to go with it through all subsequent operations of digestion—distributing disease-force to every atom of matter in the alimentary canal.

After remaining in the stomach till it has become well incorporated with the various fluids of that organ, the content of the stomach is then passed to the duodenum where it is joined with the pancreatic juice and the bile from the liver, which organ is the seat of numerous diseases, and is always liable to produce very impure or defective bile. Then, if there be purulent secretions from ulcers along the intestinal tract, we must conclude that, in the assimilative process or second stage of digestion, the chyle produced from such a mass must carry diseased force with it through the lacteals into the thoracic duct, and thence into the right vena cava. Thence, mixed with and becoming a part of the blood, it is conveyed through the heart to the lungs, back to the heart, thence with all its defects and morbid forces to every organ and tissue to replace worn-out tissue cells, to build up new tissue to wasted organs, to supply heat, sustain vital force, and strengthen the organism, and to enable the lymphatic system to eliminate effete matter from the organization.

Diseased blood is taken to the kidneys, and, with the urine, different mineral salts are secreted in solution, which when in improper condition precipitate, in the pelvis of the kidneys, concretions which pass to the urinary bladder and form gravel. In other cases, the mineral salts separate from the blood and form similar concretions in the synovial membrane, giving rise to the well-known arthritic rheumatism, gout, and bone diseases.

From this sketch of the process of digestion by diseased organs, it is easy for the reader to understand the definition of the common term, bad blood, and why it is bad. This material is carried to the connective tissues, where a certain portion is used in the formation of defective or diseased tissue, and a large part is unfit, and is placed with the effete matter to be ejected from the system, hence the feeble lymphatic vessels are over-worked, and engorgement, delays, and stoppages are the result.

Let us suppose that one of these small vessely which conveys material to the skin to be carried off by the perspiration becomes stopped ; the heavy charged fluid then presses forward and pushes the obstruction toward the skin, and by affinity it adds more and more until it becomes too large to pass further. The obstruction then becomes equal to a foreign body ; it also becomes a nucleus which attracts other morbid substances irritating to the adjacent tissues, when by the process of inflammation it becomes a receptacle for effete matter; and it soon becomes an abscess, and may be called a pimple, pustule, boil or carbuncle, according to its size and character. When it has increased in size, become pointed, has broken through the surface, and emptied its contents, it often becomes a continued running sore, but generally gets well, giving place to another; though, in cases of excessive general derangement, nature comes to the rescue, and by shutting off the appetite lessens the supply.

While much of the external surface is thus deranged a similar process is affecting the internal organs, the more fluid matters being pushed with great force upon the mucous membrane of the bowels, washing off the epithelium, making a catarrhal sore, and so flooding the digestive tract with fluid matter that diarrhœa is the result, which gives temporary relief, but is soon followed by other serious consequences. If these obstructions form in the periosteum or spongy portions of the bone, we have various bone diseases; if in the finger or toe, we have a felon; if in the hip, a hip-joint disease; if in the root of a tooth (we know the consequence) a toothache. In other cases it is different. These small collections of matter tend to lose their water and become semi-solid and not irritating to the adjacent tissues; they may become encysted and remain solid for an indefinite time, without their presence being observed. Any organ or tissue may be the seat of these deposits, but the lungs, being poorly supplied with nerves, in the parts most remote from the large air-passages, are generally the first to be invaded by these bodies. In the final result of their enlargement and breaking down there is little chance for the pus to escape, and, in order to find an exit, it must destroy more tissue than in the more external organs.

With this hasty review of the deleterious and depressing effects of a diseased condition of the digestive organs on other parts of the system, our picture is incomplete without some reference to disease-producing agents from without. There is perhaps no animal or class of animals on earth that live in more constant violation of the natural laws of health and comfort than the genus homo.

The habitual use of the fermented and distilled liquors and tobacco I leave to the temperance societies, but ask you when eating badly fried meats, fish, or cakes, fried in burning, smoking, blazing grease, to pause and think for a moment of the burning sore, itching, swollen, suppurating gland, erysipelas, putrid, cancerous, and acrid sores which are among the provings of *Carbo-animalis* before you take this carbonized animal oil into the stomach to add to other irritating substances and secretions for further destructive work on the system.

Omitting much that might be said of other articles of solid food, I will invite your attention to the modern and extensive use of ice-water, which chills the stomach, stops digestion and secretion of natural pepsin, and causes the delayed ingesta to ferment and become irritating! The vital force comes to the rescue with an increased volume of blood to warm the chilled membrane, and is again repulsed by another supply of ice-water, till this repeated action and reaction results in acute, sub-acute, or chronic gastritis and many other evils too numerous to mention in this article. It is also my object to call attention to the too extensive use of the various mineral waters, and to their subsequent effects. Let us recall the effects of some of the mineral constituents; for example, see the workings of Sulphur, how deeply it invades every organ, tissue, vital and mental faculty with its innumerable morbid symptoms and tortures from the time of inception to the hour of final dissolution. Think of the cramps of Magnesia; the rotten-smelling feet of Silicea; the anæmia from the excessive use of Iron; the glandular disturbance of the Calcareas, and many others. The great excess of these materials in solution added to the already sufficient supply taken with the natural food tends to further over-work and irritate the organs of digestion, assimiliation, and elimination, and furnish food for all the above mentioned morbid processes and many more.

I wish to call the attention of the profession to the impropriety of recommending their patients to resorts for strong mineral waters, instead of waters to which chemical analysis gives the least or no mineral elements at all.

Let them have water as perfectly pure as possible to wash the excess of mineral salts from the tissues in solution, and instruct them as to the pernicious effects of the very popular vegetable tonics, which are so extensively used by all classes of people and are recommended by so many physicians.

I do not claim that any of these statements are new to the older members of the Association, but, since the medical profession generally is drifting to surgery and specialties requiring surgery, to the neglect of the medical treatment of these important organs, I wish to impress on the younger members of the Association the sufficiency of medical treatment of this branch of practice; and that *The Organon* is not "old and out of date," as was asserted to me by a distinguished modern homœopathist. The old materia medica, which has been our reliance for so many years, still gives us, without reconstruction abundant symptomatic matter; and *The Organon* and *Chronic Diseases* will furnish ample instructions for a successful treatment of the diseases of the digestive organs for many generations yet unborn.

May the International Hahnemanian Association and Post-Graduate School of Homœopathics live and keep alive the sacred teaching of *The Organon* till that period yet hidden in the womb of time when mankind will cease to be afflicted.

